# Supplementary material for: A Critical View of the Application of the APEX Software (Aqueous Photochemistry of Environmentally-Occurring Xenobiotics) to Predict Photoreaction Kinetics in Surface Freshwaters
Source: Molecules. 2019 Dec 18;25(1):9. doi: 10.3390/molecules25010009 (PMC7017383; doi:10.3390/molecules25010009)
Supplement: Supplementary file 1 [file molecules-25-00009-s001.zip › 3.2.4_gcc-4.4.0.zip!/3.2.4_gcc-4.4.0/3.2.4_gcc-4.4.0/doc/octave/html/interpreter/Concatenating-Strings.html]

Concatenating Strings - Untitled


Next: Conversion of Numerical Data to Strings,
Up: Creating Strings


---

#### 5.3.1 Concatenating Strings

It has been shown above that strings can be concatenated using matrix notation
(see Strings, Character Arrays). Apart from that, there are several
functions to concatenate string objects: `char`,
`strvcat`, `strcat` and `cstrcat`. In addition, the general
purpose concatenation functions can be used: see cat,
horzcat and vertcat.

- All string concatenation functions except `cstrcat`
  convert numerical input into character data by taking the corresponding ASCII
  character for each element, as in the following example:

  ```
            char([98, 97, 110, 97, 110, 97])
                 => ans =
                   banana
  ```

  - `char` and `strvcat`
    concatenate vertically, while `strcat` and `cstrcat` concatenate
    horizontally. For example:

    ```
              char("an apple", "two pears")
                   => ans =
                     an apple
                     two pears
              
              strcat("oc", "tave", " is", " good", " for you")
                   => ans =
                     octave is good for you
    ```

    - `char` generates an empty row in the output
      for each empty string in the input. `strvcat`, on the other hand,
      eliminates empty strings.

      ```
                char("orange", "green", "", "red")
                     => ans =
                       orange
                       green
                
                       red
                
                strvcat("orange", "green", "", "red")
                     => ans =
                       orange
                       green
                       red
      ```

      - All string concatenation functions except `cstrcat` also accept cell
        array data (see Cell Arrays). `char` and
        `strvcat` convert cell arrays into character arrays, while `strcat`
        concatenates within the cells of the cell arrays:

        ```
                  char({"red", "green", "", "blue"})
                       => ans =
                         red
                         green
                  
                         blue
                  
                  strcat({"abc"; "ghi"}, {"def"; "jkl"})
                       => ans =
                         {
                           [1,1] = abcdef
                           [2,1] = ghijkl
                         }
        ```

        - `strcat` removes trailing white space in the arguments (except
          within cell arrays), while `cstrcat` leaves white space untouched. Both
          kinds of behavior can be useful as can be seen in the examples:

          ```
                    strcat(["dir1";"directory2"], ["/";"/"], ["file1";"file2"])
                         => ans =
                           dir1/file1
                           directory2/file2
                    
                    cstrcat(["thirteen apples"; "a banana"], [" 5$";" 1$"])
                         => ans =
                           thirteen apples 5$
                           a banana        1$
          ```

          Note that in the above example for `cstrcat`, the white space originates
          from the internal representation of the strings in a string array
          (see Character Arrays).

— Built-in Function: **char** (x)  
— Built-in Function: **char** (x, ...)  
— Built-in Function: **char** (s1, s2, ...)  
— Built-in Function: **char** (cell\_array)  
> Create a string array from one or more numeric matrices, character
> matrices, or cell arrays. Arguments are concatenated vertically.
> The returned values are padded with blanks as needed to make each row
> of the string array have the same length. Empty input strings are
> significant and will concatenated in the output.
>
> For numerical input, each element is converted
> to the corresponding ASCII character. A range error results if an input
> is outside the ASCII range (0-255).
>
> For cell arrays, each element is concatenated separately. Cell arrays converted through
> `char` can mostly be converted back with `cellstr`.
> For example,
>
> ```
>           char ([97, 98, 99], "", {"98", "99", 100}, "str1", ["ha", "lf"])
>                => ["abc    "
>                    "       "
>                    "98     "
>                    "99     "
>                    "d      "
>                    "str1   "
>                    "half   "]
> ```
>
>
>
>
> **See also:** strvcat, cellstr.

— Built-in Function: **strvcat** (x)  
— Built-in Function: **strvcat** (x, ...)  
— Built-in Function: **strvcat** (s1, s2, ...)  
— Built-in Function: **strvcat** (cell\_array)  
> Create a character array from one or more numeric matrices, character
> matrices, or cell arrays. Arguments are concatenated vertically.
> The returned values are padded with blanks as needed to make each row
> of the string array have the same length. Unlike `char`, empty
> strings are removed and will not appear in the output.
>
> For numerical input, each element is converted
> to the corresponding ASCII character. A range error results if an input
> is outside the ASCII range (0-255).
>
> For cell arrays, each element is concatenated separately. Cell arrays converted through
> `strvcat` can mostly be converted back with `cellstr`.
> For example,
>
> ```
>           strvcat ([97, 98, 99], "", {"98", "99", 100}, "str1", ["ha", "lf"])
>                => ["abc    "
>                    "98     "
>                    "99     "
>                    "d      "
>                    "str1   "
>                    "half   "]
> ```
>
>
>
>
> **See also:** char, strcat, cstrcat.

— Function File: **strcat** (s1, s2, ...)  
> Return a string containing all the arguments concatenated
> horizontally. If the arguments are cells strings, `strcat`
> returns a cell string with the individual cells concatenated.
> For numerical input, each element is converted to the
> corresponding ASCII character. Trailing white space is eliminated.
> For example,
>
> ```
>           s = [ "ab"; "cde" ];
>           strcat (s, s, s)
>                => ans =
>                   "ab ab ab "
>                   "cdecdecde"
> ```
>
> ```
>           s = { "ab"; "cde" };
>           strcat (s, s, s)
>                => ans =
>                   {
>                     [1,1] = ababab
>                     [2,1] = cdecdecde
>                   }
> ```
>
>
>
>
> **See also:** cstrcat, char, strvcat.

— Function File: **cstrcat** (s1, s2, ...)  
> Return a string containing all the arguments concatenated
> horizontally. Trailing white space is preserved. For example,
>
> ```
>           cstrcat ("ab   ", "cd")
>                => "ab   cd"
> ```
>
> ```
>           s = [ "ab"; "cde" ];
>           cstrcat (s, s, s)
>                => ans =
>                   "ab ab ab "
>                   "cdecdecde"
> ```
>
>
>
>
> **See also:** strcat, char, strvcat.
